# *Pseudomonas aeruginosa* LPS or Flagellin Are Sufficient to Activate TLR-Dependent Signaling in Murine Alveolar Macrophages and Airway Epithelial Cells

**DOI:** 10.1371/journal.pone.0007259

**Published:** 2009-10-06

**Authors:** Eloïse Raoust, Viviane Balloy, Ignacio Garcia-Verdugo, Lhousseine Touqui, Reuben Ramphal, Michel Chignard

**Affiliations:** 1 Unité de Défense Innée et Inflammation, Institut Pasteur, Paris, France; 2 INSERM U874, Paris, France; 3 Department of Medicine, University of Florida, Gainesville, Florida, United States of America; Columbia University, United States of America

## Abstract

**Background:**

The human lung is exposed to a large number of airborne pathogens as a result of the daily inhalation of 10,000 liters of air. Innate immunity is thus essential to defend the lungs against these pathogens. This defense is mediated in part through the recognition of specific microbial ligands by Toll-like receptors (TLR) of which there are at least 10 in humans. *Pseudomonas aeruginosa* is the main pathogen that infects the lungs of cystic fibrosis patients. Based on whole animal experiments, using TLR knockout mice, the control of this bacterium is believed to occur by the recognition of LPS and flagellin by TLRs 2,4 and 5, respectively.

**Methodology/Principal Findings:**

In the present study, we investigated *in vitro* the role of these same TLR and ligands, in alveolar macrophage (AM) and epithelial cell (EC) activation. Cellular responses to *P. aeruginosa* was evaluated by measuring KC, TNF-α, IL-6 and G-CSF secretion, four different markers of the innate immune response. AM and EC from WT and TLR2, 4, 5 and MyD88 knockout mice for were stimulated with the wild-type *P. aeruginosa* or with a mutant devoid of flagellin production.

**Conclusions/Significance:**

The results clearly demonstrate that only two ligand/receptor pairs are necessary for the induction of KC, TNF-α, and IL-6 synthesis by *P. aeruginosa*-activated cells, *i.e.* TLR2,4/LPS and TLR5/flagellin. Either ligand/receptor pair is sufficient to sense the bacterium and to trigger cell activation, and when both are missing lung EC and AM are unable to produce such a response as were cells from MyD88^−/−^ mice.

## Introduction

*Pseudomonas aeruginosa* is an opportunist Gram-negative bacterium that is a frequent cause of acute pneumonia in patients who are being mechanically ventilated [1]. More notoriously, *P. aeruginosa* is also the main pathogen in cystic fibrosis, infecting 70% of the patients at an early age and contributing to the chronic lung destruction responsible for mortality. In fact, *P. aeruginosa* infection rarely occurs in healthy hosts due to efficient clearance of the pathogen by the innate immune response [2], [3], [4].

As a primary interface between pathogens and the host, epithelial cells (EC) lining the mammalian airways and the alveolar surface area are a crucial site for innate immune responses [5], [6], [7], [8]. Also on the first line of the pulmonary defense against invading pathogens are alveolar macrophages (AM), which are mobile and capable of phagocytosis [9], [10], [11]. Thus, once *P. aeruginosa* enters the airways, there is direct encounter with these two cell types, which initiates a series of inducible host immune responses directed to bacterial eradication. This response includes the release of cytokines and chemokines to attract phagocytic cells to the site of infection [12]. Thus, efficient clearance of *P. aeruginosa* is considered to rely on the recognition of the pathogen by AM and EC, which mount intracellular signaling pathways responsible for triggering an innate response for host defense [2].

To accomplish this defense, the host makes use of Toll-like receptors (TLR) which are homologues of the *Drosophilia* Toll protein, and which recognize conserved microbial structures, or pathogen-associated molecular patterns (PAMP) [13], [14], [15], [16]. At present, 12 murine TLR and 10 human homologues have been cloned [17] and their ligand profile has been partially determined as reviewed [18], [19]. *P. aeruginosa* expresses numerous PAMPs [20], among which are lipopolysaccharide (LPS) [21] and flagellin [22]. LPS is a glycolipid that constitutes the major portion of the outermost membrane of Gram-negative bacteria [23], while flagellin is a protein that arranges itself in a hollow cylinder to form the filament bacterial flagellum [24]. Using a mouse experimental model of *P. aeruginosa*-induced pneumonia, our laboratory has recently shown that the sensing of LPS by TLR4 and possibly TLR2 (as the atypical LPS of *P. aeruginosa* has been reported to be detected by the latter [25], [26]), or of flagellin likely by TLR5 can effectively defend the lung from *P. aeruginosa* infection but that the absence of response by both results in hypersusceptibility to this infection [27]. This observation raised the question whether AM and EC were the effective cells of the TLR-mediated host innate immune response to *P. aeruginosa* infection? To address this question, we investigated *in vitro* the activation of these two cell types in the context of the LPS/TLR 2,4 and flagellin/TLR5 interactions. Cellular responses to *P. aeruginosa* were evaluated by measuring KC, TNF-α, IL-6 and G-CSF secretion, four different markers of the innate immune response observed in our previous *in vivo* study in which TLR2,4 have been implicated in the release of KC and TNF-α, but not of IL-6 and G-CSF [28].

## Methods

### Bacterial strains and growth conditions

The *P. aeruginosa* strain PAK, a widely studied strain of *P. aeruginosa*, was originally obtained from S. Lory (Harvard University, Boston, MA) [28]. This strain of *P. aeruginosa* is known to express a full complement of virulence factors, including pili, flagella, type II secreted enzymes, type III secreted exoenzymes S, T, and Y, exotoxin A, elastase and phospholipase and has a smooth LPS belonging to serotype 6. Δ*fliC* is a non-motile derivative of PAK in which the *fliC* gene encoding flagellin was deleted [29]. These strains were grown overnight in Luria Bertoni (LB) broth, diluted 100-fold, transferred to fresh medium, and grown for 4 h to late exponential/early stationary phase. The culture was centrifuged at 3000 g for 15 min, and the cell pellet was washed twice with cold PBS. The pellet was suspended in one-tenth the original volume in PBS and the OD_600 nm_ was adjusted to give the approximate desired inocula. The inoculum concentrations were verified by serial 10-fold dilutions of the bacterial suspensions and plating on LB agar [30]. To ensure that bacterial growth was identical for both strains of *P. aeruginosa*, in a parallel experiment a given concentration of each strain was incubated 4 hours at 37°C in LB and the OD_600 nm_ was measured at the end of the incubation period.

### Mouse strains

Males and females from several mouse strains were used for the experiments. TLR2^−/−^, TLR4^−/−^, TLR5^−/−^ and MyD88^−/−^ mice were a kind gift from S. Akira (Osaka University, Osaka, Japan) and were backcrossed eight times with C57BL/6J to ensure a similar genetic background [31]. Double-knockout TLR2^−/−^ and TLR4^−/−^ (TLR2,4^−/−^) mice were generated by breeding TLR2^−/−^ mice and TLR4^−/−^ mice, and TLR4,5^−/−^ mice were obtained in the same way. C57BL/6J mice from which these mice were derived were used as control mice. These latter mice were purchased from Charles River Laboratories (L'Arbresle, France) and were used at ∼8 wk of age. Mice were fed normal mouse chow and water *ad libitum* and were reared and housed under standard conditions with air filtration.

### Ethics Statement

Mice were cared for in accordance with Pasteur Institute guidelines in compliance with European animal welfare regulations, and all animal studies were approved by the Pasteur Institute Safety Committee in accordance with French and European guidelines.

### Primary culture of murine alveolar macrophages

Mouse AM were isolated as described [32]. Briefly, mice were killed by an intraperitoneal injection of a lethal dose of pentobarbital sodium (Céva Santé Animale, Libourne, France), tracheas were cannulated, and lungs were washed several times with 0.7 mL PBS to provide 10 mL of broncho-alveolar lavages (BAL). Resident AM were collected from the BAL and centrifuged at 400 g for 15 min at 4°C. Pellets then were pooled in 1 mL lysate buffer (composed of NH_4_Cl 8.83 g/L, KHCO_3_ 6.628 g/L, and EDTA(Na)_2_ 1.25 g/L in H_2_O) for 5 min to eliminate red blood cells and then 10 mL PBS were added to stop the reaction. The solution was centrifuged again (400 g, 15 min, 4°C). Cell pellets were resuspended in RPMI 1640 medium (Invitrogen, San Francisco, CA) supplemented with 2 mM L-glutamine (Invitrogen) and 10% Fetal Calf Serum (FCS) (Hyclone, Logan, UT), counted and dispensed into 96-well tissue culture plates (TPP, Trasadingen, Switzerland). Cells (10^5^ macrophages/200 µl/well) were incubated in a 5% CO_2_ humidified atmosphere for 1 hour at 37°C for adhesion, before stimulation. Cells were then washed and incubated with different concentrations of bacteria suspended in the same medium, as indicated in the figure legends, and centrifuged (80 g, 4 min, 4°C) to increase the adherence between cells and bacteria, as well as to ensure similar contact between wild-type and non-motile *P. aeruginosa*. Conditioned media (250 µL) were collected after 4 hours of incubation, centrifuged (400 g, 5 min, 4°C) and stored at –20°C. In all cases, experiments were performed with a pool of cells collected from several mice, as indicated in the figure legends and all time points were performed in triplicate.

### Isolation, purification and culture of epithelial cells

EC were isolated from the mouse lung according to a protocol adapted from Corti *et al.* [33], and previously used by Warshamana *et al.* [34] and Bortnick *et al.* [35]. Briefly, mice were euthanasized by an intraperitoneal injection of a lethal dose of pentobarbital sodium, tracheas were cannulated, and lungs were washed three times with 0.7 mL PBS to remove alveolar macrophages. Lungs were then perfused with 10–20 mL PBS supplemented with 1% antibiotics solution (Invitrogen; composed of penicillin and streptomycin, plus antifungal amphotericin B) injected into the heart ventricle until they were cleared of blood. This was done using a 10-mL syringe and a 21-gauge needle (Terumo, Leuven, Belgium). Two milliliters of dispase (BD Biosciences, Bedford, MA) were instilled into the lungs with a 2-mL syringe (Terumo) through the cannulated trachea, which was then ligated. Lungs were then removed en bloc and placed in a sterile tube containing 1 mL of dispase and incubated for 45 minutes at room temperature for enzymatic digestion to occur.

Lung tissue was separated from large bronchi by mechanical means with forceps and teased apart in 4 mL of F12K nutrient mixture (Kaighn's modification, Invitrogen) with 0.01% DNase I (Sigma, St Louis, MO) and 1% antibiotics solution on a 6-well plate (BD Falcon, Franklin Lakes, NJ). The cell isolate was filtered successively through nylon mesh filters of sizes 100, 40 (BD Falcon) and finally 30 µm (Miltenyi Biotec, Bergisch Gladbach, Germany). The cell suspension thus obtained was centrifuged at 400 g for 10 minutes at 4°C to collect the cell pellet, which was resuspended in F12K supplemented with 2% FCS, 1% antibiotics solution and 2 mM L-glutamine.

Cells were purified and cultured by a slight modification of the methods described by Corti *et al.* [33], and by Dobbs *et al.* [36]. Approximately 3×10^7^ cells of the crude cell suspension in 2% FCS were plated on 100-mm tissue-culture-treated dishes (BD Falcon) coated with rat anti-mouse anti-CD 45 (42 µg) and rat anti-mouse anti-CD 16/32 (16 µg) (both from Pharmingen, San Diego, CA) diluted in 5 mL PBS, and incubated for 2 hours in a humidified, 5% CO2 incubator at 37°C, in order to allow the attachment of leukocytes, monocytes and dendritic cells. The unattached epithelial cells were collected by panning the dish and the suspension was centrifuged as described before. The cell pellet was resuspended in F12K supplemented with 10% FCS, 1% antibiotics solution and 2 mM L-glutamine. Viability was determined by trypan blue exclusion, before cells were plated. Cell suspensions were plated on a 96-well plate (5×10^4^ cells/well), coated beforehand with fibronectin (Sigma) (5 µg/mL of F12K). Cells were grown for one week until confluence. The medium was renewed once or twice during that period to remove remaining red blood cells and primary culture debris. Then cells were stimulated with different concentrations of bacteria suspended in the same medium (without antibiotics), as indicated in the figure legends, and centrifuged (80 g, 4 min, 4°C) to increase the adherence between cells and bacteria, as well as to ensure similar contact between wild-type and non-motile *P. aeruginosa*. Conditioned media (300 µL) were collected after 4 hours of incubation, centrifuged (400 g, 5 min, 4°C), stored at −20°C and all time points were performed in triplicate.

The purity of the cell monolayer was evaluated following cell detachment by trypsin (5 min, 37°C) (Sigma) and staining with anti-cytokeratin (Progen Biotechnik, Heidelberg, Germany) and anti-ZO-1 (Invitrogen Immunodetection, San Francisco, CA) antibodies, by microscopic immunofluorescence and by FACS analysis. Cells were 96.4±1.1% positive for the cytokeratin staining, 81.7±12.7% positive for the ZO-1 staining, and 92.6±4.2% positive for the cytokeratin/Z0-1 double staining, as measured by FACS, indicating their epithelial phenotype. To better identify these epithelial cells, we examined their phenoptypes more precisely. Freshly isolated cells were 95% alveolar type II cells as judged by the presence of Nile red-positive vacuoles, in agreement with previous reports using a similar protocol for cell purification and Nile red staining [35], [37]. After one week of culture, *i.e.* at the time of bacterial challenge, the percentage of cells containing Nile red-positive vacuoles was again evaluated. Only five percent of the cells were alveolar type II cells. The percentage of Clara cells, nonciliated bronchiolar cells, was also evaluated by nitro blue tetrazolium (NBT) staining [38], [39] at that time point. Although, 70–90% of bronchiolar cells in the mouse lung are Clara cells [40], none of our adherent cells were positive for NBT staining. Finally, we checked the presence of type I cells after a 1 week of culture by examining the expression of a specific marker, namely aquaporin 5. Using an appropriate anti-aquaporin 5 antibody (Alomone, Jerusalem, Israel), we observed intracellular labeling in 93.8±5.6% (6 different observations) of the total cell population, indicating that the majority of the cells were type I alveolar epithelial cells, in agreement with a previous report [41].

### Cytotoxicity and Total Cell Number Assay

Cytotoxicity was measured with the CytoTox 96 Nonradioactive Cytotoxicity assay (Promega, Madison, WI), following the manufacturer's protocol. The CytoTox 96 assay measures the lactate dehydrogenase (LDH) activity released from cells by the generation of a red colored product [42]. *P. aeruginosa* had no cytotoxic effect on the cells, at the concentrations and exposure time used.

This assay was also adapted to quantify the total cell number per well, at the end of the incubation periods. Briefly, cell samples were lysed by incubation with 200 µL per well of Lysis Solution (9% (v/v) Triton X-100 in water) at 37°C for 45 minutes. Fifty µl supernatant were then transferred to the enzymatic assay plate and mixed with 50 µl Substrate Mix. The enzymatic LDH activity was allowed to proceed for 30 minutes, protected from light, and the Stop Solution was added. The plate was read at 490 nm using an ordinary ELISA plate reader. The number of cells present is directly proportional to the absorbance values, which represent LDH activity [43].

### Reagents

LPS from *E. coli*, serotype 0111:B4 (TLRgrade) (Alexis Biochemicals, Axxora, San Diego, CA) was used to stimulate both cell types. Recombinant *P. aeruginosa* flagellin was prepared as described before [44]. Phorbol 12-myristate 13-acetate (PMA, Sigma) was used in combination with ionomycine (Calbiochem, Darmstadt, Germany) as described before [45]. It is of note that all these reagents were used as positive control to check the phenotype of the cell preparations. In the case of the comparison of TLR2,4−/− *vs* wild-type macrophages, LPS from *P. aeruginosa*, serotype 10 (purified by gel-filtration chromatography; Sigma-L8643) was also used, with exactly the same pattern of response as for *E. coli* LPS (data not shown), indicating that effects were comparable. Nonetheless, as *P. aeruginosa* LPS is far less efficient than *E. coli* LPS in terms of cell activation, and is far less purified and as such susceptible to display false positive response, we choose to employ the latter throughout the experiments.

### KC, TNF-α, IL-6 and G-CSF ELISA Assay

Murine KC, TNF-α, IL-6 and G-CSF concentrations in cell culture supernatants were determined using DuoSet ELISA assay kits (R&D Systems, Minneapolis, MN) with TMB peroxidase substrate (KPL, Washington, D.C.).

### Statistical Analysis

Cytokine levels were expressed as the mean±SEM. Differences between groups were assessed for statistical significance using the ANOVA test, followed by the Fisher test. A value of p<0.05 was considered statistically significant.

## Results

### Alveolar Macrophages

#### Stimulation of WT and TLR2,4^−/−^ alveolar macrophages by *P. aeruginosa*

We first tested whether TLR2 and TLR4 are critical for the secretion of KC, TNF-α, IL-6 and G-CSF induced by *P. aeruginosa* infection, by comparing the response of WT and TLR2,4^−/−^ AM isolated from mouse BAL.

**Figure 1:**
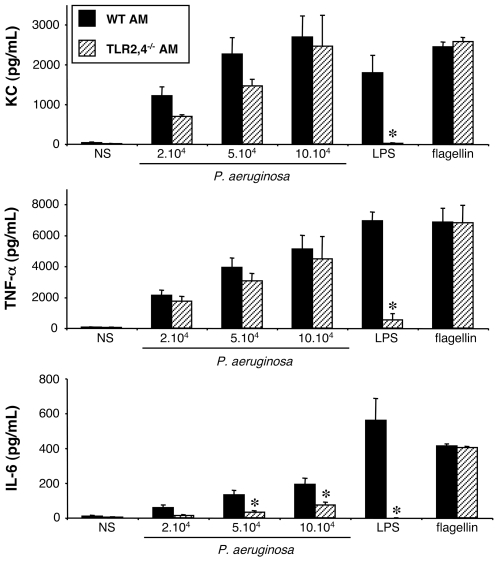
Effect of TLR2 and 4 expression on KC, TNF-α and IL-6 synthesis by alveolar macrophages challenged with *P. aeruginosa*. Alveolar macrophages (AM) collected from wild-type (WT) and TLR2,4^–/–^ mice were infected for 4 h with increasing concentrations (number of bacteria/well) of *P. aeruginosa* wild-type strain PAK. *NS*, non-stimulated cells cultured with medium only. LPS (1 µg/mL) and flagellin (20 ng/mL) were used as control agonists to stimulate the cells. Values represent means±SEM of three to five experiments performed in triplicate. *, *p*<0.05 when compared with the corresponding WT values.

At the basal state (NS), cells from both strains of mice showed only a limited production of these mediators (Fig. 1). Exposure of WT AM for 4 hours to increasing concentrations of the wild-type PAK strain of *P. aeruginosa* elicited increasing productions of KC, IL-6 and TNF-α. LPS from *E. coli* and flagellin purified from *P. aeruginosa* also stimulated the production of these mediators, and acted as a positive control for the AM activation. Nevertheless, AM from both mouse strains did not produce any G-CSF (data not shown) neither in response to bacteria, nor to LPS or flagellin.

AM from TLR2,4^−/−^ mice released a large amount of TNF-α, and KC which was slightly less than that released by WT AM, but not significantly different (Fig. 1). By contrast, the pattern of IL-6 production by TLR2,4^−/−^ AM was clearly different. Indeed, above 5×10^4^ bacteria per well, the production was significantly decreased compared to that of WT AM. Finally, purified LPS did not induce the production of any of the cytokines, reflecting the lack of expression of TLR4 by these cells. On the contrary, flagellin still induced the production of the three cytokines, indicating that it was still recognized by AM.

These first results show that the recognition of LPS as it is expressed by live *P. aeruginosa* plays a limited role in the synthesis of TNF-α and KC, but was important for the synthesis of IL-6 by AM.

#### Stimulation of WT and MyD88^−/−^ alveolar macrophages by *P. aeruginosa*

**Figure 2:**
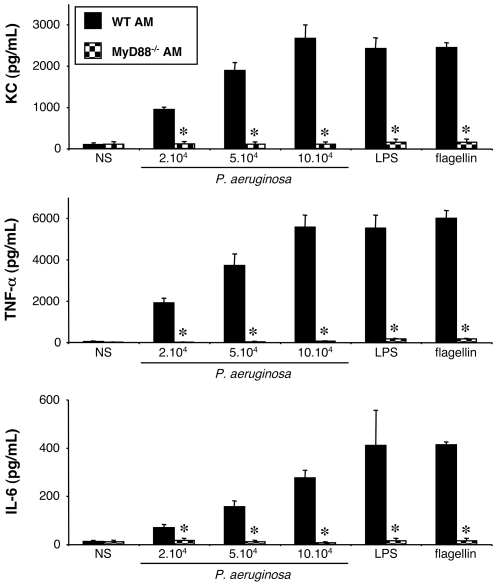
Effect of MyD88 expression on KC, TNF-α and IL-6 synthesis by alveolar macrophages challenged with *P. aeruginosa*. Alveolar macrophages (AM) collected from WT and MyD88^–/–^ mice were infected for 4 h with increasing concentrations (number of bacteria/well) of *P. aeruginosa* wild-type strain PAK. *NS*, non-stimulated cells cultured with medium only. LPS (1 µg/mL) and flagellin (20 ng/mL) were used as control agonists to stimulate the cells. Values represent means±SEM of three to five experiments performed in triplicate. *, *p*<0.05 when compared with the corresponding WT values.

The ligand recognition by all TLRs, except TLR3, initiates a cascade of signaling pathways involving the small adaptor protein MyD88 (46). To determine whether *P. aeruginosa*-induced KC and TNF-α synthesis by AM is mediated by a TLR, the response of AM isolated from MyD88^−/−^ mice was compared to the response of WT AM. As a result, MyD88 deficient AM did not produce any of the studied cytokines (KC, TNF-α, and IL-6), whatever the bacterial concentration used (Fig. 2). Purified LPS and flagellin did not activate these cells either. To verify that MyD88^−/−^ AM were nonetheless able to produce the investigated cytokines, we used a combination of PMA and ionomycin [45] as a positive control. PMA (2 µg/mL) and ionomycin (5 µM) induced in MyD88^−/−^ AM, a 4.5-fold, 18-fold and 3-fold secretion of KC, TNF-α and IL-6, respectively. Those activations were similar to the ones obtained with WT AM (data not shown).

These results show that *i*) no other receptor working independently of MyD88 is involved in the synthesis of KC, TNF-α and IL-6 by *P. aeruginosa*-challenged under our experimental conditions, and that *ii*) at least one TLR, other than TLR2 and TLR4, signaling *via* MyD88, is certainly implicated. As, in an *in vivo* experimental model of *Pseudomonas* lung infection, we previously demonstrated the recognition of flagellin by the host [46], we hypothesized that TLR5 expressed by AM could be involved in the recognition of *P. aeruginosa* not mediated by TLR2,4.

#### Stimulation of WT alveolar macrophages by *P. aeruginosa* and a Δ*fliC* mutant strain

**Figure 3:**
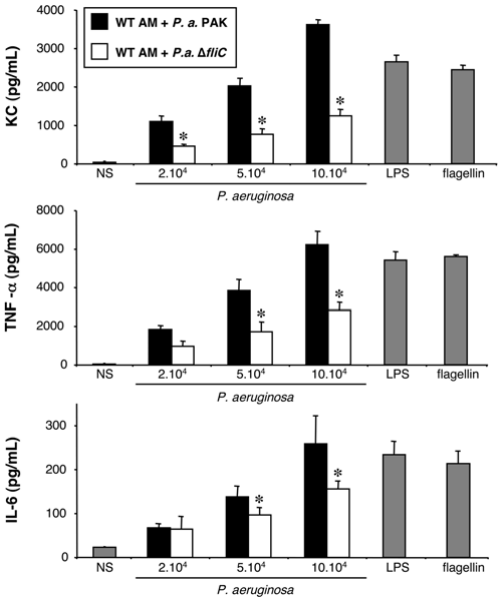
Effect of flagellin expression on KC, TNF-α and IL-6 synthesis by alveolar macrophages challenged with *P. aeruginosa*. Alveolar macrophages (AM) collected from WT mice were infected for 4 h with increasing concentrations (number of bacteria/well) of *P. aeruginosa* wild-type strain PAK or with a mutant devoid of flagellin production, Δ*fliC*. *NS*, non-stimulated cells cultured with medium only. LPS (1 µg/mL) and flagellin (20 ng/mL) were used as control agonists to stimulate the cells. Values represent means±SEM of three to five experiments performed in triplicate. *, *p*<0.05 when Δ*fliC* values are compared with the corresponding PAK values.

To explore the importance of the TLR5/flagellin pathway, we employed two isogenic strains of *P. aeruginosa*, the wild-type strain and a Δ*fliC* mutant strain (flagellin mutant) to stimulate WT AM. When stimulated with the Δ*fliC* mutant strain, AM produced significantly less KC, TNF-α, and IL-6, in comparison with the wild-type bacterial strain (Fig. 3).

These data indicate that *P. aeruginosa* flagellin is sensed by AM and triggers their activation. TLR5, the receptor of flagellin, is thus possibly involved in the response of AM to *P. aeruginosa*. Nonetheless, this possible flagellin-TLR5 interaction does not fully account for the activation of AM, as the absence of flagellin does not lead to a complete lack of cytokine synthesis. Since *in vivo* an inability to control *P. aeruginosa* pulmonary infections is observed in the absence of recognition of both LPS and flagellin [27], we therefore examined the combined effect of these two ligands under *in vitro* situation.

#### Stimulation of WT and TLR2,4^−/−^ alveolar macrophages by the Δ*fliC* mutant strain

**Figure 4:**
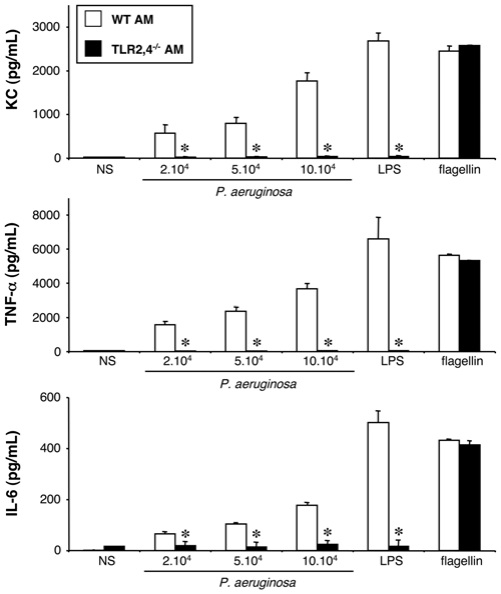
Effect of TLR2,4 and flagellin expression on KC, TNF-α and IL-6 synthesis by alveolar macrophages challenged with *P. aeruginosa*. Alveolar macrophages (AM) collected from WT and TLR2,4^–/–^ mice were infected for 4 h with increasing concentrations (number of bacteria/well) of a mutant *P. aeruginosa* devoid of flagellin production, Δ*fliC*. *NS*, non-stimulated cells cultured with medium only. LPS (1 µg/mL) and flagellin (20 ng/mL) were used as control agonists to stimulate the cells. Values represent means±SEM of three to five experiments performed in triplicate. *, *p*<0.05 when compared with the corresponding WT values.

TLR2,4^−/−^ AM were stimulated by the Δ*fliC* mutant strain of *P. aeruginosa*. In response to this stimulation, TLR2,4^−/−^ AM did not produce KC, TNF-α, nor IL-6 (Fig. 4).

Taken together, these data demonstrate that the recognition of *P. aeruginosa* by AM is mediated by flagellin, certainly *via* TLR5, but also by LPS *via* TLR2,4.

#### Stimulation of WT and TLR4,5^−/−^ alveolar macrophages by *P. aeruginosa*

**Figure 5:**
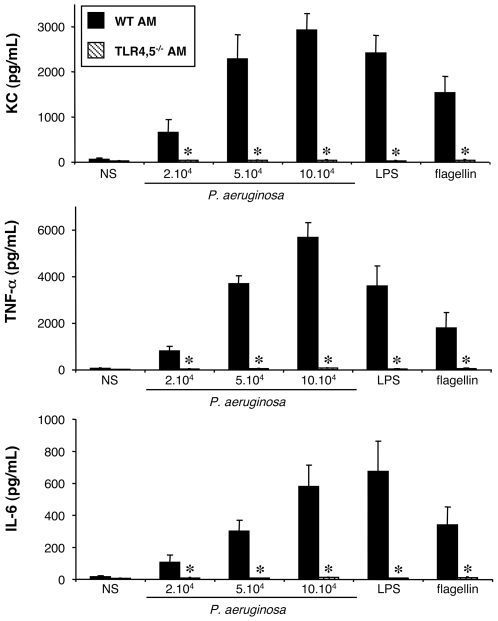
Effect of TLR4 and 5 expression on KC, TNF-α and IL-6 synthesis by alveolar macrophages challenged with *P. aeruginosa*. Alveolar macrophages (AM) collected from WT and TLR4,5^–/–^ mice were infected for 4 h with increasing concentrations (number of bacteria/well) of *P. aeruginosa* wild-type strain PAK. *NS*, non-stimulated cells cultured with medium only. LPS (1 µg/mL) and flagellin (20 ng/mL) were used as control agonists to stimulate the cells. Values represent means±SEM of three to five experiments performed in triplicate. *, *p*<0.05 when compared with the corresponding WT values.

Finally, to confirm the role of TLR5 in the recognition of *P. aeruginosa*, we used TLR4,5^−/−^ AM, which were stimulated by the wild-type strain of *P. aeruginosa* (Fig. 5). As expected, productions of KC, TNF-α, and IL-6 were completely abolished in these cells.

These results confirm the previous data obtained with the Δ*fliC* mutant strain, and show that the recognition of flagellin by AM is indeed mediated by TLR5.

The entire study thus demonstrates that only two ligand/receptor pairs are necessary for the activation of AM by *P. aeruginosa* in terms of KC, TNF-α, and IL-6 production, R2,4/LPS and TLR5/flagellin. Either one is sufficient and when both are missing AM are unable to produce the studied cytokines.

### Epithelial Cells

#### Stimulation of WT and MyD88^−/−^ epithelial cells by *P. aeruginosa*

To better understand the mechanisms involved in the recognition of *P. aeruginosa* by the lung, we investigated the response of EC to this pathogen, since like AM, they are known to be on the first line of defense against invading pathogens. Primary EC purified from the lungs of mice were therefore stimulated with the same bacteria as for AM and the same cytokines were assayed.

**Figure 6:**
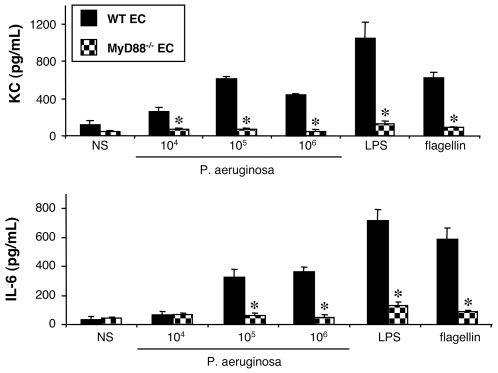
Effect of MyD88 expression on KC and IL-6 synthesis by lung epithelial cells challenged with *P. aeruginosa*. Lung epithelial cells (EC) collected from WT and MyD88^–/–^ mice were infected for 4 h with increasing concentrations (number of bacteria/well) of *P. aeruginosa* wild-type strain PAK. *NS*, non-stimulated cells cultured with medium only. LPS (1 µg/mL) and flagellin (20 ng/mL) were used as control agonists to stimulate the cells. Values represent means±SEM of three to five experiments performed in triplicate. *, *p*<0.05 when compared with the corresponding WT values.

At the basal state (NS), cells from both WT and MyD88^−/−^ mice showed only a limited production of these mediators (Fig. 6). Exposure of EC from WT mice to increasing concentrations of the wild-type strain of *P. aeruginosa* for 4 hours elicited increasing amounts of KC and IL-6, but not of TNF-α or G-CSF (data not shown). Purified LPS and flagellin also stimulated the production of KC and IL-6, and acted as a control for the EC activation.

To determine whether the EC response to *P. aeruginosa* infection was mediated by a TLR, cells isolated from MyD88^−/−^ mice were stimulated by the wild-type strain, in comparison with WT EC. Like for AM, MyD88-deficient EC did not produce KC or IL-6, whatever the bacterial concentration used (Fig. 6). Purified LPS and flagellin did not activate these cells either. To verify that MyD88^−/−^ EC were nonetheless able to produce these two cytokines, we used the same combination of PMA and ionomycin as for AM as a positive control. PMA plus ionomycin added to MyD88^−/−^ EC induced a 8-fold and 3-fold secretion of KC and IL-6, respectively. These secretions were similar to those obtained with the WT EC (data not shown).

These results show that i) at least one TLR signaling *via* MyD88 is involved in the recognition of *P. aeruginosa* by EC, and that ii) no MyD88-independent pathway is involved in the secretion of KC and IL-6.

#### Stimulation of WT and TLR2,4^−/−^ epithelial cells by *P. aeruginosa* and the Δ*fliC* mutant strain

**Figure 7:**
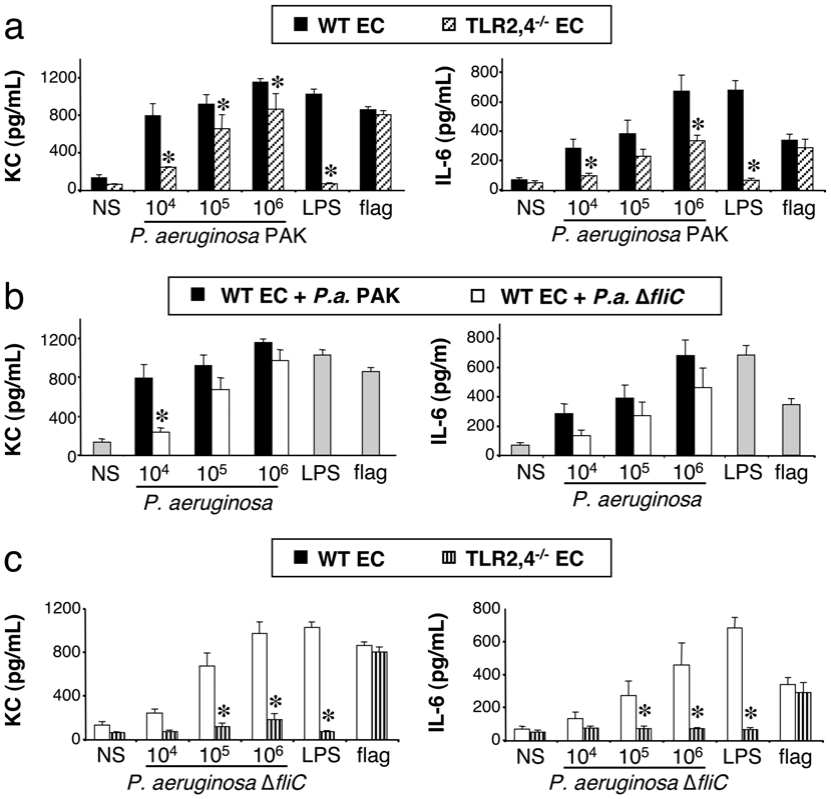
Effect of TLR2,4 and flagellin expression on KC and IL-6 synthesis by lung epithelial cells challenged with *P. aeruginosa*. Lung epithelial cells (EC) collected from mice were infected for 4 h with increasing concentrations (number of bacteria/well) of *P. aeruginosa*. Different combinations were used, *i.e.*, WT and TLR2,4^−/−^ mice cells stimulated with the wild-type bacteria strain PAK (7a); WT mice cells stimulated with the wild type bacteria strain PAK and its mutant Δ*fliC* (7b); WT and TLR2,4^−/−^ mice cells stimulated with the Δ*fliC* mutant (7c). *NS*, non-stimulated cells cultured with medium only. LPS (1 µg/mL), and flagellin (20 ng/mL) were used as control agonists to stimulate the cells. Values represent means±SEM of three to five experiments performed in triplicate. *, *p*<0.05 when compared with either the corresponding WT mice or wild-type PAK strain values.

To search for the TLR that was implicated, we followed the same experimental approach with EC as we did with AM. TLR2,4^−/−^ EC produced a large amount of KC and IL-6 in response to wild-type bacteria, though slightly reduced compared to WT EC (Fig. 7a). As seen with AM, purified LPS did not induce the production of any of the cytokines, while flagellin did, reflecting the lack of expression of TLR4 by these cells. These experiments show that the recognition of *P. aeruginosa* LPS would only play a limited role in the activation of primary EC of the mouse and support studies done on established human EC lines [47].

We next stimulated WT EC with the Δ*fliC* mutant strain, in response to which they produced approximately the same amount of KC and IL-6, in comparison with the wild-type strain (Fig. 7b). These data indicate that flagellin as it is expressed by live *P. aeruginosa* does not seem to play an important role in the activation of EC in terms of KC and IL-6 production.

To verify the implication of two pathways, as we found for AM, TLR2,4^−/−^ EC were stimulated by the Δ*fliC* mutant strain of *P. aeruginosa*. In response to this stimulation, TLR2,4^−/−^ EC did not produce any KC and IL-6 (Fig. 7c).

Taken together, these data demonstrate that *P. aeruginosa* is sensed by EC through either LPS recognition by TLR2,4, or flagellin recognition probably by TLR5.

#### Stimulation of WT and TLR4,5^−/−^ epithelial cells by *P. aeruginosa*

**Figure 8:**
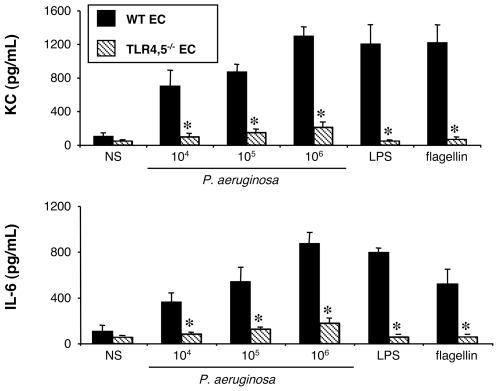
Effect of TLR4 and 5 expression on KC and IL-6 synthesis by lung epithelial cells challenged with *P. aeruginosa*. Lung epithelial cells (EC) collected from WT and TLR4,5^−/−^ mice were infected for 4 h with increasing concentrations (number of bacteria/well) of *P. aeruginosa* wild-type strain PAK. *NS*, non-stimulated cells cultured with medium only. LPS (1 µg/mL), and flagellin (20 ng/mL) were used as control agonists to stimulate the cells. Values represent means±SEM of three to five experiments performed in triplicate. *, *p*<0.05 when compared with the corresponding WT values.

Finally, to confirm that the recognition of flagellin is mediated by TLR5, we used TLR4,5^−/−^ EC, which were stimulated by wild-type *P. aeruginosa* (Fig. 8). As seen with AM, the production of KC and IL-6 were completely abolished in these cells.

These results confirm the previous data obtained with the Δ*fliC* mutant strain, and show that the recognition of flagellin by the EC is indeed mediated by TLR5.

In summary, for EC as for AM, at least two pathways are at play for the sensing of *P. aeruginosa*, and either one is sufficient for an efficient recognition of these bacteria by EC.

## Discussion

The message inferred from the present study is straightforward, *i.e.* AM and EC, the two cell types that first encounter *P. aeruginosa* during the process of lung infection, sense the bacterium and trigger an innate cellular response through a MyD88-dependent pathway and even exclusively through this pathway in the case of KC and IL-6 synthesis. It is of note that recently von Bernuth *et al*. [48] have shown that humans deficient in MyD88 have an increased susceptibility to *P. aeruginosa*. From these data one may consider the implication of MyD88-dependent but TLR-independent cascade, *i.e.* the IL-1 receptor (IL-1R) pathway [49]. However, it is unlikely that IL-1R is involved as we saw no cytokine response to challenge with this organism when the participation of certain TLR is prevented. In fact, in our hands at play are only two pairs of ligand-receptor, namely LPS-TLR2,4 and flagellin-TLR5. Moreover, we showed that these two pathways have a redundant role as suppression of only one of the two is not followed by a large significant loss of cell response, and that by contrast, when both are disrupted, there was a dramatic crippling of the responses that we examined.

These results obtained *in vitro* are in agreement with *in vivo* data from Feuillet *et al*. [50] showing that TLR4,5^−/−^ mice are hypersusceptible to lung infection by wild-type *P. aeruginosa*, and from our laboratory [27] demonstrating that TLR4 and TLR5 play major but redundant roles in controlling bacterial replication during host defense against *P. aeruginosa* pneumonia. By contrast, they differ from the data reported by Skerett *et al.* [51] showing that TLR2,4^−/−^ mice were not hypersusceptible to a *P. aeruginosa* strain lacking flagellin. As previously explained [27] this could simply be due to the use of a low bacterial load by the latter authors compared to the two formers. Nonetheless, the same group [51] reported that bone marrow cells lacking both TLR2 and TLR4 exhibited a reduced TNF-α production to heat-killed flagellated *P. aeruginosa* but no response to the heat-killed Δ*fliC* mutant. We extended their data using *in vitro* experimental conditions more closely related to the *in vivo* situation *i.e*. AM but not bone marrow-derived macrophages and live but not heat-killed bacteria. Thus, we report here on the synthesis of other mediators, KC and IL-6, the response of another cell type, EC, and the effect of another defect, TLR5 deficiency. Such a study was necessary as bone marrow-derived macrophages have been reported to be not responsive to flagellin [52], [53] and that heat-killed bacteria do not release flagellin in the medium as do live bacteria (Ramphal, unpublished observation). The remarkable point is that AM and EC behave in almost the same way in response to *P. aeruginosa*, both using TLR2,4 and TLR5 for the recognition of the bacterium and to engage an innate immune response. Indeed, Ipaf could have been implicated as this intracellular receptor has been shown to sense flagellin [54], [55], although it is implicated in the processing of immature to mature IL-1β and IL-18 [56] and not of IL-6 and KC. From the *in vivo* situation, it can be also deduced that Ipaf probably plays a minor role if any during lung infection as mice lacking both TLR4 and 5 are hypersusceptible to *P. aeruginosa*, dying within less than a day [50]. This lack of impact on the survival has been observed experimentally although Ipaf seems to be required for the early elimination of the bacterium *in vivo* [46]. Another aspect that needs to be discussed here is the involvement of the Cystic fibrosis transmembrane conductance regulator (CFTR) as a receptor of *P. aeruginosa* [57]. Indeed, Pier *et al.* have shown that CFTR may mediate the innate immune response to *P. aeruginosa* [7], [58], [59]. Thus, patients with non-functional CFTR, *i.e.* CF patients, are more susceptible to *P. aeruginosa* infection than other patients. Nevertheless, these patients develop chronic lung infections, which are different from acute infections observed in non CF patients, as bacteria undergo phenotypic changes as the clinical condition of the CF patient evolves [60], [61]. We studied here the role of TLR in a context of acute lung infection, which may explain the important impact that these receptors have in our model. Thus, even though CFTR has been shown to provide some contribution, TLR are critical for an efficient innate immune response against *P. aeruginosa*.

If qualitatively there are no differences between AM and EC as indicated above, quantitative differences are nonetheless observed. Thus, with AM the lack of expression of flagellin by *P. aeruginosa* leads to a significant reduction of KC, TNF-α and IL-6 secretion (see Fig. 3), a feature that is not so salient when EC are used. It is of note that *in vivo* the intratracheal administration of the Δ*fliC* mutant strain compared to the wild type strain of *P. aeruginosa* does not modify the production of KC, TNF-α and IL-6 measured in the BAL [48]. This suggests that *in vivo* the participation of EC in the synthesis of cytokines and chemokines would be more important than the participation of AM, which is in agreement with two recent reports demonstrating that airway EC critically regulates the innate immune response to *P. aeruginosa* [62], [63]. Another difference is that AM collected from TLR2,4^−/−^ mice synthesize very low concentrations of IL-6 compared to those recovered from AM from WT mice (see Fig. 1). However, the difference is not so pronounced with EC (see Fig. 8). These data have to be compared to those observed *in vivo* [28]. Surprisingly, there is no correlation between the *in vivo* and *in vitro* experiments when comparing mediator production by WT and TLR2,4^−/−^ mice. Indeed, TLR2,4 have been implicated in the release of KC, TNF-α, but not of IL-6 in the *in vivo* experiments [28] while in the present study TLR2,4 expressed by either AM or EC are implicated in IL-6 synthesis but only marginally in that of KC (AM and EC) and TNF-α (AM). These contradictory data poses a problem as to which cell types explain the *in vivo* data? Apparently it is neither AM nor EC. Several hypotheses can be raised such as the presence of dendritic cells, or the recruitment of polymorphonuclear neutrophils, or also a mechanism of cooperation between AM and EC. To answer this question, a thorough study will be necessary, *in vitro* by using co-culture of AM and EC, and *in vivo* by identifying the cell types producing a given mediator by immunocytochemistry. Another interesting question is the following. As TNF-α, KC, and IL-6 syntheses are all dependent on MyD88 activation and consequently NF-κB nuclear translocation, which aspect of the signaling pathway(s) triggered by *P. aeruginosa* in TLR2,4^−/−^ AM leads to TNF-α and KC and not to IL-6 formation?

One can wonder if the presently described redundant mechanism based on the recognition of LPS by TLR2,4 and of flagellin by TLR5 is operative in human cells. At least it is firmly established that human AM and EC express TLR2 and 4 and are activated by LPS. Also, both human cell populations express TLR5 and are activated by flagellin [64], [65], [66], [67], [22]. Redundancy is probably essential in humans given the fact that approximately 10% of Caucasians carry a polymorphism in the ligand-binding domain of TLR5 (stop codon) that acts in a dominant fashion to abolish flagellin signaling [68]. This polymorphism is associated with a slightly increased susceptibility to Legionnaires' disease, but otherwise the carriers are apparently not more susceptible to infection with flagellated bacteria as demonstrated with *Salmonella enterica* [69]. At the opposite end, it has been recently reported that the TLR5 mRNA expression is increased in cystic fibrosis airway EC and that, as a probable consequence, these cells almost exclusively rely upon TLR5 to sense *P. aeruginosa* [70]. This phenotype would be beneficial in the frame of the chronic *P. aeruginosa* infection observed in these patients, although one can speculate that it could also be at the origin of the inflammatory status characterizing the lung of cystic fibrosis patients.
